# Some Urgently Needed Reforms

**Published:** 1919-12-27

**Authors:** 


### HEALTH PROBLEMS IN IRELAND.

### Some Urgently Needed Reforms.

THE *Times* has recently drawn attention to some of the pressing problems in Ireland. At first sight the whole question appears to be of such a complicated nature that to find a solution seems well-nigh impossible; however, the problem must, and will, be solved. One fact stands out predominantly, viz. that the old system of Poor Law is not only obsolete, but is actually a bar to reform; it is cumbrous, expensive, and does not always provide the benefit in the quarter for which it was originated.

### THE TRAGEDIES OF IGNORANCE AND DISEASE.

The scourge of tuberculosis, which claims so many victims year by year, and which is ever present, requires a more educated control for its eradication; it is useless for a medical man to prescribe treatment when the latter cannot be carried out, or only to a very limited extent. Now it is essential that, in the absence of the requisite number of sanatoria, the tuberculous patient should be treated in his own home; but if this line of treatment is to be of any avail, both in curing or relieving the sufferer, and also in preventing the spread of the disease to any of the other members of the family, it is absolutely necessary that certain fundamental principles should be closely followed. Thus there must be fresh air and sunshine and good nourishing food. The tubercle bacillus revels in darkness and foul atmospheres, and therefore the patient should be in a room well ventilated, and so placed that the maximum amount of light can gain admittance. Few people seem to understand that it is not the quantity but the quality of the food that decides the amount of nutrition in the body; surely education of this principle should be in every curriculum of the teacher on public health. Inferior coal in the furnace means deficient steam in the engine; and this statement aptly applies to the human machine also. If the Irish people are to be contented they must first be sound in health. Now to become a healthy adult one must first be a healthy infant; therefore it is incumbent on those who make themselves responsible for that most important problem, child welfare, that the education of the masess be directed to combat superstitious ignorance. Superstition and ignorance go hand in hand, and have heavy and justifiable accusations laid to their charge. The mother demands our unceasing care, and when at last she has given to the world her loved offspring, neither should be left to the tender mercies of blind ignorance, or, only too often, wilful neglect, but be treated on rational lines.

Perhaps there is nothing which shows the retrogression of a people more than their blind faith in chance treatment of disease; surely it does not need a clever brain to realise that the causal treatment of any malady is much more scientific, and therefore efficacious, than the symptomatic. Yet every year millions of persons put their implicit trust in quack remedies, and then, when too late, wonder why they have received no real benefit; there will always exist a section of dishonest persons ever ready to impose on the credulous and unthinking, and it should be the duty of the medical profession to teach these ignorant people how they are being fooled, both in health and also in pocket.

A consideration of statistics dealing with cases suffering from mental defects does not make for encouraging reading, and it is obvious that there is a great deal of work waiting to be carried out in this region of prophylaxis. As our knowledge of the internal secretions in the body increases, especially those produced by the thyroid and pituitary glands, it is quite conceivable that this section of disease will not prove to be so insurmountable a barrier as it is at present. There is already adequate proof that some forms of mental disease can be very greatly relieved, and sometimes cured. Now the realisation of this ideal requires an efficient staff of experts, both medical and lay, with suitable hospitals specially set apart for the purpose. The time and money expended will be well justified by the increasing number of good results, bringing more and more benefit to a country which needs healthy sons and daughters, perhaps more now than ever.

### THE NEED FOR BETTER HOUSING.

Every one knows that "You cannot make a silk purse out of a sow's ear," and similarly it should be universally recognised that you cannot expect people to keep themselves clean and healthy in filthy surroundings; nor is it logical to preach cleanliness without providing the where-with-all to keep clean. As the *Times* rightly points out, there is, perhaps, no other city in the world that can produce worse slums than are to be found in Dublin, and this the principal city in Ireland

Is it not a criminal offence that, in these days of advanced civilisation, it is possible for landlords to receive profitable rents from tenants of dwellings which are hardly fit for pigs, much less human beings? The time has come when local councils must insist on a much more improved system of housing and at a reasonable rent; this necessarily involves a great financial outlay; whole areas requiring to be demolished and rebuilt. To effect these and other no less urgent reforms demands an adequate supply of trained doctors, nurses, and laymen, who will devote their united energies to the work in hand; they must be properly paid and equipped with facilities for research, for no advance is possible without experiment, and this itself cannot be carried on without the unhampered means to conduct it. It should not be impossible to organise a scheme of centralisation, say, in the principal city of each county, with branches in the smaller towns and villages responsible to the one head. As to the question of finances to support this organisation, let us remember that men and women only really appreciate what they have had to pay for, and have, therefore, been to some trouble to obtain it, and it should be quite possible to formulate a basis of contribution from every adult.

### THE ADVANCE OF DEMOCRACY.

The advance of democracy seems to be bringing in its train an almost overwhelming element of officialism, with necessarily increased expenditure, and no little irritation; surely this defeats the very object for which the true democrat is striving, viz., the betterment of the masses, in order that the latter may be able to share, to an ever greater extent, in the benefits accruing from their labour; for thus, and thus only, will contentment be established. There is no doubt that there has been a gross mismanagement of Irish affairs, and success will never attend the efforts of politicians and administrators unless they tackle this problem with an open and disinterested mind, working for the ideal that "Brotherhood is better than Creed."

